# Supplementary figures and images for: Characterization of Cover Crop Rooting Types from Integration of Rhizobox Imaging and Root Atlas Information
Source: Plants (Basel). 2019 Nov 17;8(11):514. doi: 10.3390/plants8110514 (PMC6918168; doi:10.3390/plants8110514)

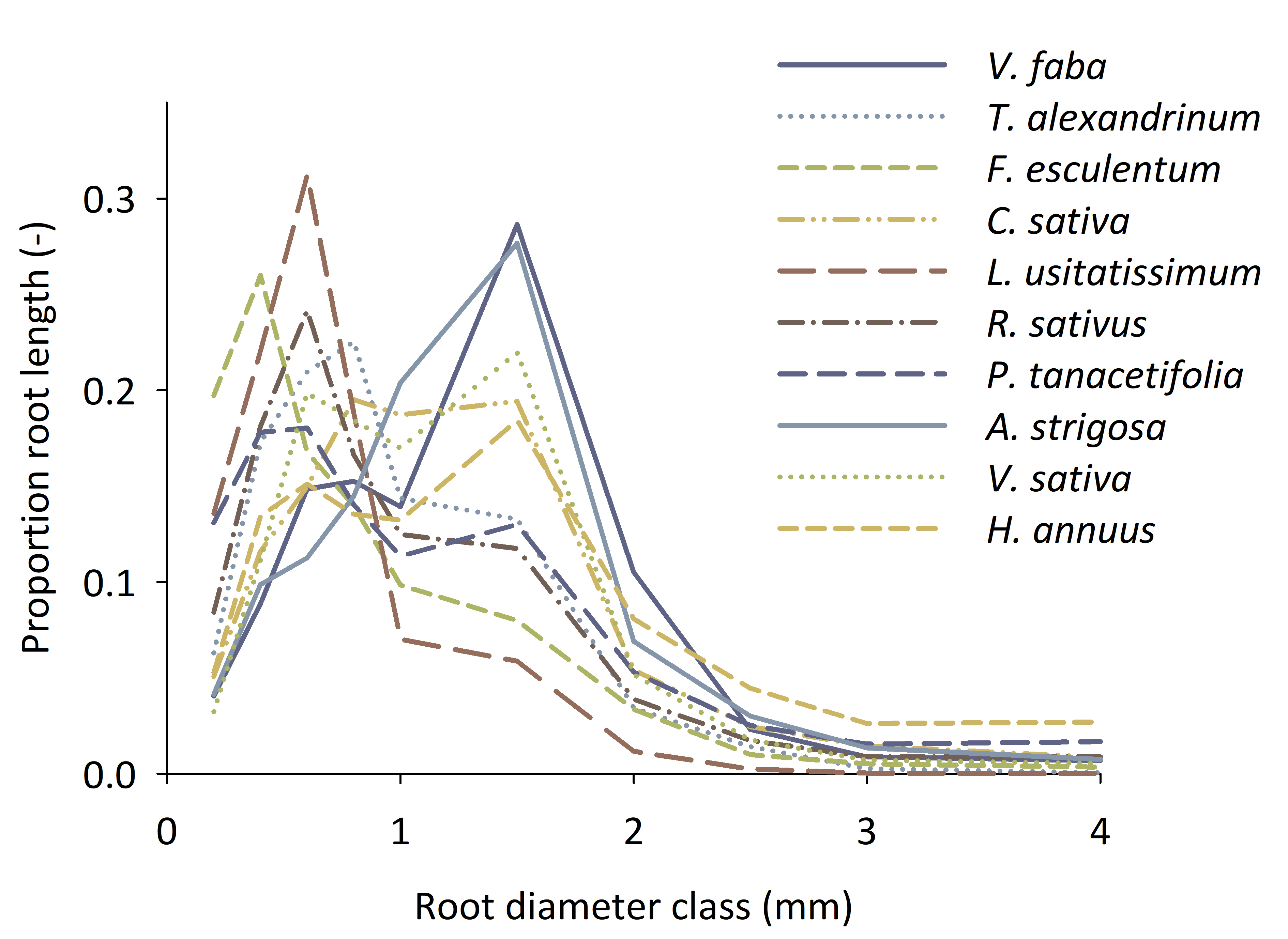

Supplement: Supplementary file 1 [file plants-08-00514-s001.jpg]
